# Antimicrobial activity of ceftolozane/tazobactam, imipenem/relebactam, and comparators against Gram-negative pathogens collected in Arabian Gulf countries: SMART 2020–2024

**DOI:** 10.3389/fcimb.2026.1827575

**Published:** 2026-05-12

**Authors:** Mark G. Wise, Irina Alekseeva, Fakhar Siddiqui, Katherine Young, Mary R. Motyl, Daniel F. Sahm

**Affiliations:** 1 IHMA, Schaumburg, IL, United States; 2 MSD, Dubai, United Arab Emirates; 3 Merck & Co., Inc., Rahway, NJ, United States

**Keywords:** ceftolozane/tazobactam, Enterobacterales, imipenem/relebactam, *Pseudomonas aeruginosa*, SMART

## Abstract

**Objectives:**

To investigate the *in vitro* susceptibility of recent clinical Enterobacterales and *Pseudomonas aeruginosa* isolates collected in the Arabian Gulf region to ceftolozane/tazobactam, imipenem/relebactam, and comparator antimicrobial agents.

**Methods:**

From 2020 to 2024, two clinical laboratories in Kuwait and one each in Qatar, the United Arab Emirates, and Oman (participated in 2022–2024 only) collected up to 250 consecutive Gram-negative isolates per year from patients with bloodstream, intra-abdominal, lower respiratory tract, and urinary tract infections. MICs were determined by CLSI broth microdilution and interpreted with 2025 EUCAST breakpoints. Most imipenem, imipenem/relebactam, and ceftolozane/tazobactam non-susceptible isolates were interrogated for their acquired β-lactamase content.

**Results:**

Ceftolozane/tazobactam was active against 85.6% of the Enterobacterales (*n* = 3,603), including 92.0% of ESBL-positive, non-CRE (non-carbapenem-resistant Enterobacterales) phenotype *Escherichia coli* and 91.6% of ESBL-positive, non-CRE phenotype *Klebsiella pneumoniae*, but was poorly active against MDR (multiple-drug resistant) isolates (21.6% susceptible). In total, 90.6% of non-*Morganellaceae* Enterobacterales (*n* = 3,421) were imipenem/relebactam-susceptible, including 95.6% of the *E. coli* and 80.7% of the *K. pneumoniae*. *Pseudomonas aeruginosa* isolates (*n* = 1,347) were highly susceptible to both ceftolozane/tazobactam and imipenem/relebactam, with 91.2% and 89.0% inhibited, respectively. Both ceftolozane/tazobactam and imipenem/relebactam retained activity against ≥70% of cefepime-resistant, ceftazidime-resistant, and piperacillin/tazobactam-resistant *P. aeruginosa*. Ceftolozane/tazobactam inhibited the greatest percentage of meropenem-resistant *P. aeruginosa* (66.9%) among comparator β-lactam antimicrobials. Molecular characterization showed that the majority of both the imipenem/relebactam- and ceftolozane/tazobactam-resistant Enterobacterales harbored the NDM metallo-β-lactamase (MBL). Most of the imipenem/relebactam-resistant *P. aeruginosa* characterized did not possess acquired β-lactamases, while the majority of those resistant to ceftolozane/tazobactam carried a variety of acquired enzymes, including MBLs (IMP, VIM, NDM) and ESBLs (VEB, GES).

**Conclusion:**

Recent clinical isolates of Enterobacterales collected in the Arabian Gulf region were highly susceptible to imipenem/relebactam, while both imipenem/relebactam and ceftolozane/tazobactam exhibited excellent activity against *P. aeruginosa.* However, the incidence of MBLs in the region remains a significant concern for future therapeutic strategies.

## 1 Introduction

Antimicrobial resistance (AMR) among Gram-negative bacteria poses a critical threat to public health and underscores the need for robust surveillance programs able to detect resistance patterns and inform treatment guidelines. The Middle East, and in particular the countries bordering the Arabian Gulf, have historically reported high rates of AMR in clinical settings (Aly and Balkhy, 2012; Zowawi et al., 2013). A recent One Health meta-analysis focusing on countries in the Middle East, including those in the Arabian Gulf area, highlighted the high rates of resistance to β-lactam agents, often attributable to the spread of ESBLs and carbapenemases (Alkheraije, 2024). In many Gulf Cooperation Council countries, including Kuwait, Qatar, Saudi Arabia, and the United Arab Emirates, high rates of multidrug-resistant (MDR) Enterobacterales have been noted, as 22%–29% are ESBL-producing, with even higher rates among intensive care unit and urinary tract infection isolates (Hadi et al., 2023).

Ceftolozane/tazobactam combines an antipseudomonal cephalosporin with tazobactam, a traditional β-lactamase inhibitor of many Ambler class A β-lactamases, although it does not inhibit KPC-type carbapenemases and some GES- and PER-type β-lactamases (Ortiz de la Rosa et al., 2019). Imipenem/relebactam is a combination of a carbapenem with a newer β-lactamase inhibitor, relebactam, which also inhibits class A enzymes, notably including KPC (Livermore et al., 2013). Ceftolozane/tazobactam was first approved by the U.S. FDA in 2014 (and by the EMA in 2015) and is indicated for complicated intra-abdominal infections (in combination with metronidazole), complicated urinary tract infections including pyelonephritis, and hospital-acquired or ventilator-associated bacterial pneumonia (in adults) (European Medicines Agency, 2015; United States Food and Drug Administration, 2019). Imipenem/relebactam was approved later (FDA 2019; EMA 2020) and is indicated for hospital-acquired or ventilator-associated pneumonia and, in patients with limited or no alternative treatment options, for complicated urinary tract and intra-abdominal infections; the European label also includes treatment of infections due to aerobic Gram-negative organisms with limited options and associated bacteremia (European Medicines Agency, 2020; United States Food and Drug Administration, 2020). In an effort to update *in vitro* susceptibility surveillance data for ceftolozane/tazobactam and imipenem/relebactam against clinical isolates of Gram-negative bacilli from Arabian Gulf countries, we evaluated the activity of these two agents and relevant comparators against isolates collected from 2020 to 2024 by clinical laboratories in Kuwait, Oman, Qatar, and the United Arab Emirates that participated in the Study for Monitoring Antimicrobial Resistance Trends (SMART) global surveillance program.

## 2 Materials and methods

### 2.1 Bacterial isolates

Two hospitals in Kuwait, one in Qatar, and one in the United Arab Emirates participated in the SMART program each year from 2020 through 2024. One hospital in Oman participated for 3 years (2022 to 2024). Each site collected consecutive, aerobic, or facultative Gram-negative isolates from bloodstream, intra-abdominal, lower respiratory tract, and urinary tract infections. The program stipulates that only one isolate per patient per species per year should be retained. All isolates were sent to one of two central laboratories (IHMA, Monthey, Switzerland, or IHMA, Schaumburg, IL, USA), where species identity was confirmed using MALDI-TOF mass spectrometry (Bruker Daltonics, Billerica, MA, USA). A brief summary of the demographic and clinical characteristics associated with all isolates that were a part of this study is provided in the **Supplementary Data** (**Supplementary Table 1**).

### 2.2 Antimicrobial susceptibility and interpretation

The CLSI reference broth microdilution method (CLSI, 2024) was utilized to determine antimicrobial susceptibility, and minimum inhibitory concentrations (MICs) were interpreted employing 2025 EUCAST breakpoints (European Committee on Antimicrobial Susceptibility Testing, 2025). Relebactam and tazobactam were tested at a fixed concentration of 4 µg/mL, in combination with doubling dilutions of imipenem and ceftolozane, respectively. For ceftolozane/tazobactam, a range of 0.12 to 16 mg/L was tested, while for imipenem/relebactam, a range of 0.03 to 16 mg/L was tested. Imipenem/relebactam susceptibility was analyzed for non-*Morganellaceae* Enterobacterales (NME) only since EUCAST does not publish breakpoints for imipenem/relebactam against the *Morganellaceae* (including the genera *Proteus*, *Providencia*, and *Morganella*). Members of this family display increased resistance to imipenem by a mechanism independent of β-lactamase production, and relebactam does not improve the antimicrobial activity of imipenem against this group (CLSI, 2025).

An ESBL-positive, non-CRE (non-carbapenem-resistant Enterobacterales) phenotype was defined for isolates of *Escherichia coli* and *Klebsiella pneumoniae* as those testing with a ceftriaxone MIC ≥2 mg/L and an ertapenem MIC ≤0.5 mg/L. The ESBL-positive, non-CRE designation was not extended to isolates of *Klebsiella oxytoca* and *Proteus mirabilis* as too few isolates met these criteria for meaningful analysis. For Enterobacterales, an MDR phenotype was defined as resistance (by EUCAST criteria) to ≥3 sentinel agents (amikacin, cefepime, colistin, levofloxacin, meropenem, and piperacillin/tazobactam), while difficult-to-treat resistance (DTR) was defined as resistance (EUCAST) to all tested β-lactams (including ceftazidime, cefepime, ceftriaxone, imipenem, meropenem, ertapenem, piperacillin/tazobactam), as well as fluoroquinolones (levofloxacin) (Magiorakos et al., 2011; Kadri et al., 2018). For *P. aeruginosa*, MDR was defined as an isolate testing as resistant (EUCAST) to ≥3 sentinel drugs: amikacin, aztreonam, cefepime, colistin, meropenem, levofloxacin, and piperacillin/tazobactam. DTR *P. aeruginosa* was defined as an isolate testing as resistant by EUCAST to all β-lactams (including aztreonam, cefepime, ceftazidime, imipenem, meropenem, piperacillin/tazobactam) and fluoroquinolones (levofloxacin). For both Enterobacterales and *P. aeruginosa*, the definition of DTR excluded antimicrobial susceptibility testing results for ceftolozane/tazobactam, imipenem/relebactam, and ceftazidime/avibactam.

### 2.3 Statistical analysis

The Cochran–Armitage test (Armitage, 1955) was used to assess country-specific linear trends in annual percentage susceptible values from 2020 to 2024 for Kuwait, Qatar, and the United Arab Emirates, and from 2022 to 2024 for Oman. A two-tailed *p*-value <0.05 was considered statistically significant.

### 2.4 Screening for β-lactamase genes

NME isolates (excluding *Serratia* spp.) testing with imipenem/relebactam MIC values of ≥2 mg/L, imipenem MIC values of ≥2 mg/L (in 2020–2022) or ≥4 mg/L (in 2023–2024), and *P. aeruginosa* isolates testing with imipenem or imipenem/relebactam MIC values of ≥4 mg/L were screened for acquired β-lactamase genes. Also qualifying for β-lactamase characterization were Enterobacterales with ceftolozane/tazobactam MIC values of ≥4 mg/L and *P. aeruginosa* isolates with ceftolozane/tazobactam MIC values of ≥8 mg/L. For Enterobacterales collected in 2020–2022, published multiplex PCR assays were used to screen for β-lactamase genes as described previously (Lob et al., 2015; Nichols et al., 2016). For *P. aeruginosa* collected in 2020–2023 and Enterobacterales in 2023 only, isolates were characterized by short-read whole-genome sequencing to a targeted coverage depth of 100×, as previously described (Estabrook et al., 2021), and analyzed using the CLC Genomics Workbench (Qiagen, Venlo, Netherlands). The detection of β-lactamase genes was performed by comparison to the ResFinder database (Bortolaia et al., 2020). For all qualifying isolates collected in 2024, molecular characterization was carried out using whole genome sequencing by PromethION 2 Integrated (Oxford Nanopore Technologies, Oxford, United Kingdom) using the R10.4.1 flow cell. Base calling was performed using the super-accurate model (dorado 7.6.7). Assembly was conducted using the EPI2ME isolates workflow version 1.4.1. Antimicrobial resistance markers were identified using AbritAMR (Sherry et al., 2023). The deduced amino acid sequences of identified β-lactamase genes were extracted so that variants could be determined using the NCBI Bacterial Antimicrobial Resistance Reference Gene Database (BioProject 313047). In total, 488 Enterobacterales and 387 P*. aeruginosa* were molecularly characterized.

## 3 Results

Overall, 85.6% of all Enterobacterales isolates collected in Arabian Gulf countries, including 90.6% of *E. coli* isolates, 75.7% of *K. pneumoniae* isolates, 93.5% of *Citrobacter* spp., 87.0% of *Enterobacter* spp., 96.2% of the *Morganellaceae*, 95.7% of *Serratia* spp., and 85.2% of *Klebsiella* spp. (other than *K. pneumoniae*), were susceptible to ceftolozane/tazobactam (**Table 1**). Regarding *E. coli* and *K. pneumoniae* exhibiting the ESBL-positive, non-CRE phenotype, 92.0% and 91.6% were inhibited (i.e., interpreted as susceptible EUCAST criteria) by ceftolozane/tazobactam, respectively, values approximately 13 percentage points higher than the traditional standard of care antimicrobial for the region, piperacillin/tazobactam. Ceftolozane/tazobactam was poorly active against MDR Enterobacterales (21.6% inhibited) and completely inactive against DTR Enterobacterales.

**Table 1:** Antimicrobial susceptibility of clinical isolates of Enterobacterales and *Pseudomonas aeruginosa* collected in the Arabian Gulf region, 2020–2024.

Organism group or phenotype	*n*	% Susceptible													
		C/T	IMR	IPM^a^, ^b^	MEM	ETP	CZA	CAZ^b^	FEP^b^	CRO	TZP^b^	ATM^b^	LVX^b^	AMK	CST
Enterobacterales	3,603	85.6	NA	89.7	90.5	88.3	91.9	55.5	62.4	56.6	78.9	NA	60.3	92.6	85.7
NME	3,421	85.0	90.6	89.9	90.0	87.8	91.5	54.1	61.0	55.2	77.9	NA	61.0	92.5	90.2
*E. coli*	1,440	90.6	95.6	95.3	95.3	93.8	95.6	46.5	54.5	48.2	84.0	NA	50.5	97.0	99.7
ESBL non-CRE^c^	661	92.0	100	100	100	100	100	3.2	14.4	0.0	82.8	NA	32.5	96.8	99.7
* K. pneumonia*	1,259	75.7	80.7	79.6	79.6	76.9	83.1	49.6	53.9	51.9	67.6	NA	59.1	84.5	92.8
ESBL non-CRE^c^	322	91.6	100	100	100	100	100	4.7	9.9	0.0	78.0	NA	47.8	98.1	98.4
* Citrobacter* spp.	93	93.5	100	100	100	98.9	100	83.9	91.4	83.9	84.9	NA	89.2	98.9	100
* Enterobacter* spp.	231	87.0	97.4	97.0	97.0	90.9	97.4	72.7	85.7	69.3	81.4	NA	84.4	98.3	71.0
* Klebsiella* spp.^d^	162	85.2	95.7	95.1	95.7	93.2	96.9	63.0	80.2	63.0	74.1	NA	81.5	96.9	98.8
* Morganellaceae*	182	96.2	NA	NA	98.9	98.4	98.9	80.2	88.5	81.9	96.7	NA	45.6	93.4	NA
* Serratia* spp.	186	95.7	98.4	97.8	98.9	97.3	98.9	91.4	91.4	86.6	91.4	NA	91.9	95.7	NA
CRE^e^	339	0.9	5.6	0.3	1.8	0.9	16.2	0.9	0.9	0.9	0.6	3.9	3.5	44.5	75.5
MDR	537	21.6	40.0	36.3	37.1	31.7	46.2	2.6	2.0	2.8	4.3	NA	1.9	54.4	78.4
DTR	295	0.0	1.4	0.0	0.0	0.0	11.9	0.0	0.0	0.0	0.0	NA	0.0	41.0	75.6
*P. aeruginosa*	1,347	91.2	89.0	69.6	67.1	NA	88.0	67.8	70.2	NA	63.7	66.6	79.8	93.2	99.7
CAZ-resistant	434	73.5	72.1	41.2	35.9	NA	63.1	0.0	15.0	NA	4.6	19.6	60.4	81.8	99.5
FEP-resistant	401	72.1	70.1	39.7	33.9	NA	61.3	8.0	0.0	NA	4.7	14.0	53.9	79.1	99.5
TZP-resistant	489	77.9	73.2	42.5	35.8	NA	69.1	15.3	21.9	NA	0.0	20.4	59.9	83.0	99.6
MEM-resistant	254	66.9	46.1	6.7	0.0	NA	53.1	22.8	22.4	NA	12.6	15.0	40.9	77.2	100
MDR	240	62.8	53.4	22.6	14.3	NA	49.2	12.4	4.5	NA	1.9	15.8	28.6	68.0	99.2
DTR	94	47.9	25.5	0.0	0.0	NA	25.5	0.0	0.0	NA	0.0	0.0	0.0	64.9	100

C/T, ceftolozane/tazobactam; IMR, imipenem/relebactam; IPM, imipenem; MEM, meropenem; ETP, ertapenem; CZA, ceftazidime/avibactam; CAZ, ceftazidime; FEP, cefepime; CRO, ceftriaxone; TZP, piperacillin/tazobactam; LVX, levofloxacin; AMK, amikacin; CST, colistin; NME, non-*Morganellaceae* Enterobacterales; ESBL, extended-spectrum β-lactamase; CRE, carbapenem-resistant Enterobacterales; MDR, multidrug-resistant; DTR, difficult-to-treat resistant. NA, not tested or MIC breakpoint not available.

^a^ The results combine % susceptible, increased exposure values for *Morganellaceae*, and % susceptible values for non-*Morganellaceae* Enterobacterales.

^b^ For IPM, CAZ, FEP, TZP, ATM, and LVX against *P. aeruginosa*, the results represent “% Susceptible, Increased Exposure” as defined in the EUCAST guidelines.

^c^ ESBL non-CRE phenotype was defined by an isolate testing with a CRO MIC ≥2 mg/L and an ETP MIC ≤0.5 mg/L.

^d^ Excludes *K. pneumoniae.*

^e^ CRE defined as an Enterobacterales isolate resistant to imipenem and/or meropenem by EUCAST criteria.

Imipenem/relebactam was highly active, inhibiting 90.6% of NME and 100% of ESBL-positive non-CRE phenotype *E. coli* and *K. pneumoniae*. Meropenem (90.5% susceptible), ceftazidime/avibactam (91.9%), and amikacin (92.6%) also inhibited very high percentages of Enterobacterales isolates, while cefepime, ceftazidime, ceftriaxone, piperacillin/tazobactam, and levofloxacin were less active, inhibiting <80% of the isolates. Colistin inhibited 85.7% of all Enterobacterales, but 90.2% of the NME, as it is inactive against members of the *Morganellaceae* family (Falagas and Kasiakou, 2005). Amikacin and colistin were the only agents inhibiting >50% of the MDR Enterobacterales and >40% of the DTR Enterobacterales.

Ceftolozane/tazobactam (91.2% susceptible) and imipenem/relebactam (89.0%) were both active against the full collection of *P. aeruginosa* isolates (*n* = 1,347) and retained activity against approximately 73%–78% (ceftolozane/tazobactam) and 70%–73% (imipenem/relebactam) of ceftazidime-resistant, cefepime-resistant, and piperacillin/tazobactam-resistant isolates (**Table 1**). Against meropenem-resistant isolates, 66.9% and 46.1% were ceftolozane/tazobactam- and imipenem/relebactam-susceptible, respectively. Both ceftolozane/tazobactam (62.8%) and imipenem/relebactam (53.4%) inhibited >50% of the MDR *P. aeruginosa*, but <50% of the *P. aeruginosa* identified as DTR. Ceftazidime/avibactam was also active against the full collection of *P. aeruginosa* (88% susceptible); however, among the drug-resistant phenotypes, ceftolozane/tazobactam inhibited greater percentages of isolates, including >10 percentage points more ceftazidime-resistant, cefepime-resistant, and meropenem-resistant MDR isolates and >20 percentage points more DTR isolates. Amikacin and colistin were also quite potent against the full collection of *P. aeruginosa*, each inhibiting >90% of the isolates. However, despite their excellent *in vitro* activity, it is important to note the practical limitations associated with colistin and amikacin use in treating Gram-negative infections. Colistin is associated with dose-limiting nephrotoxicity and neurotoxicity, which may necessitate dose reduction or discontinuation, thus compromising therapeutic efficacy. Similarly, amikacin use is limited by well-documented adverse effects, including nephrotoxicity, ototoxicity, and neurotoxicity, particularly with prolonged therapy or higher doses. EUCAST only publishes bracketed colistin and amikacin (systemic infections) MIC breakpoints with a warning against the use of these agents without additional therapeutic measures. The CLSI does not publish susceptible MIC breakpoints for colistin against any Gram-negative pathogen.

**Figure 1:**
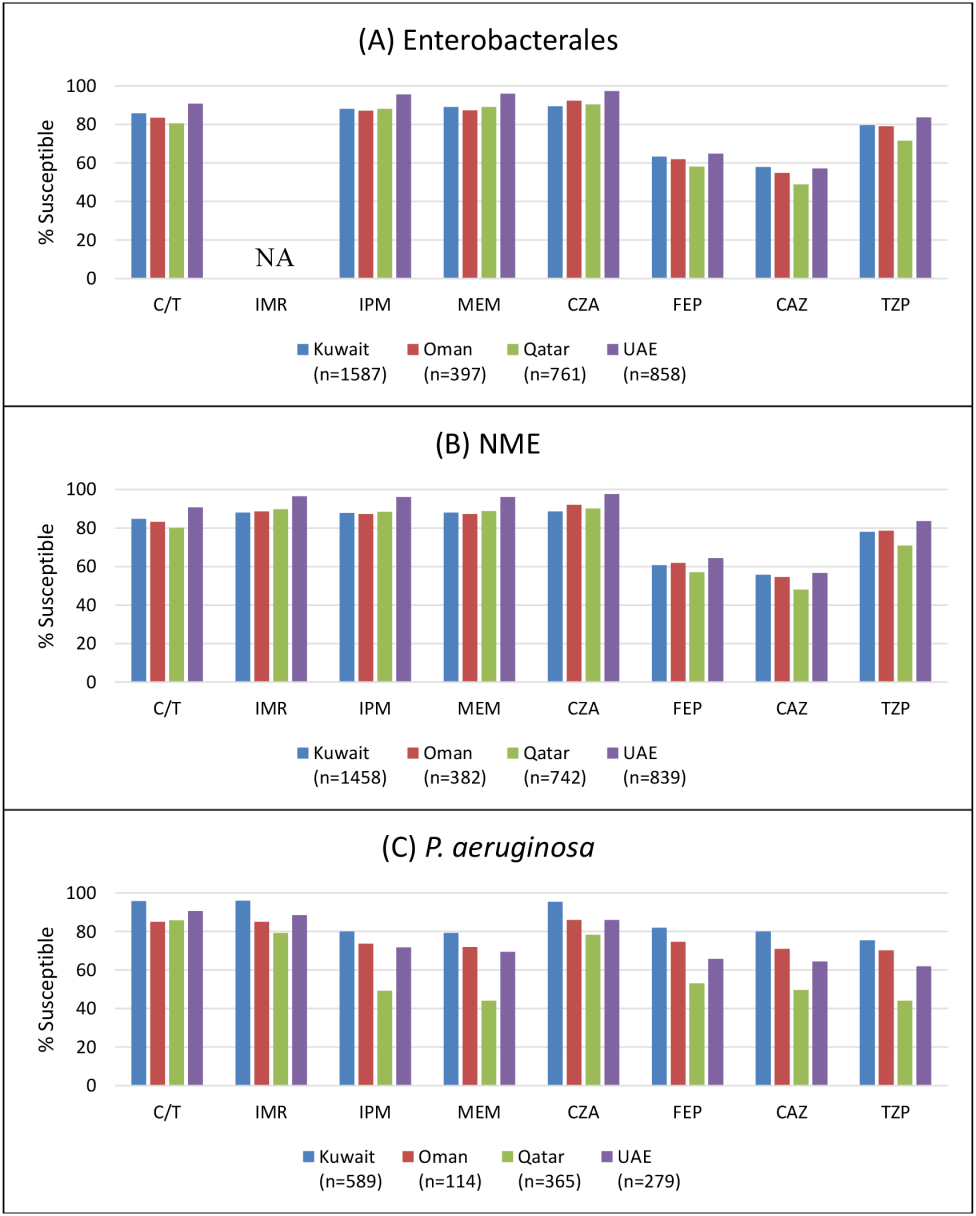
Susceptibility of **(A)** Enterobacterales, **(B)** non-*Morganellaceae* Enterobacterales (NME), and **(C)** *Pseudomonas aeruginosa* to β-lactam antimicrobials by country. C/T, ceftolozane/tazobactam; IMR, imipenem/relebactam; IPM, imipenem; MEM, meropenem; CZA, ceftazidime/avibactam; CAZ, ceftazidime; FEP, cefepime; TZP, piperacillin/tazobactam. For IPM against Enterobacterales, the results combine % susceptible, increased exposure values for *Morganellaceae*, and % susceptible values for non-*Morganellaceae*. For IPM, CAZ, FEP, and TZP against *P. aeruginosa*, the results represent “% Susceptible, Increased Exposure.” NA, not applicable (no EUCAST breakpoints for IMR against Enterobacterales). Percent susceptible values presented in this figure are provided in **Supplementary Table 2**.

**Figure 1** illustrates the percentages of isolates interpreted as susceptible to ceftolozane/tazobactam, imipenem/relebactam, and β-lactam comparators by country of collection. In general, among the Enterobacterales, isolates from the UAE were the most susceptible to the tested agents, while those from Qatar were the least susceptible [except to meropenem (Oman the least susceptible)]. Similar results were observed considering just the NME, with ceftazidime/avibactam and imipenem/relebactam the most active agents in each country. Among *P. aeruginosa*, isolates from Kuwait were consistently the most susceptible to all agents.

**Figure 2:**
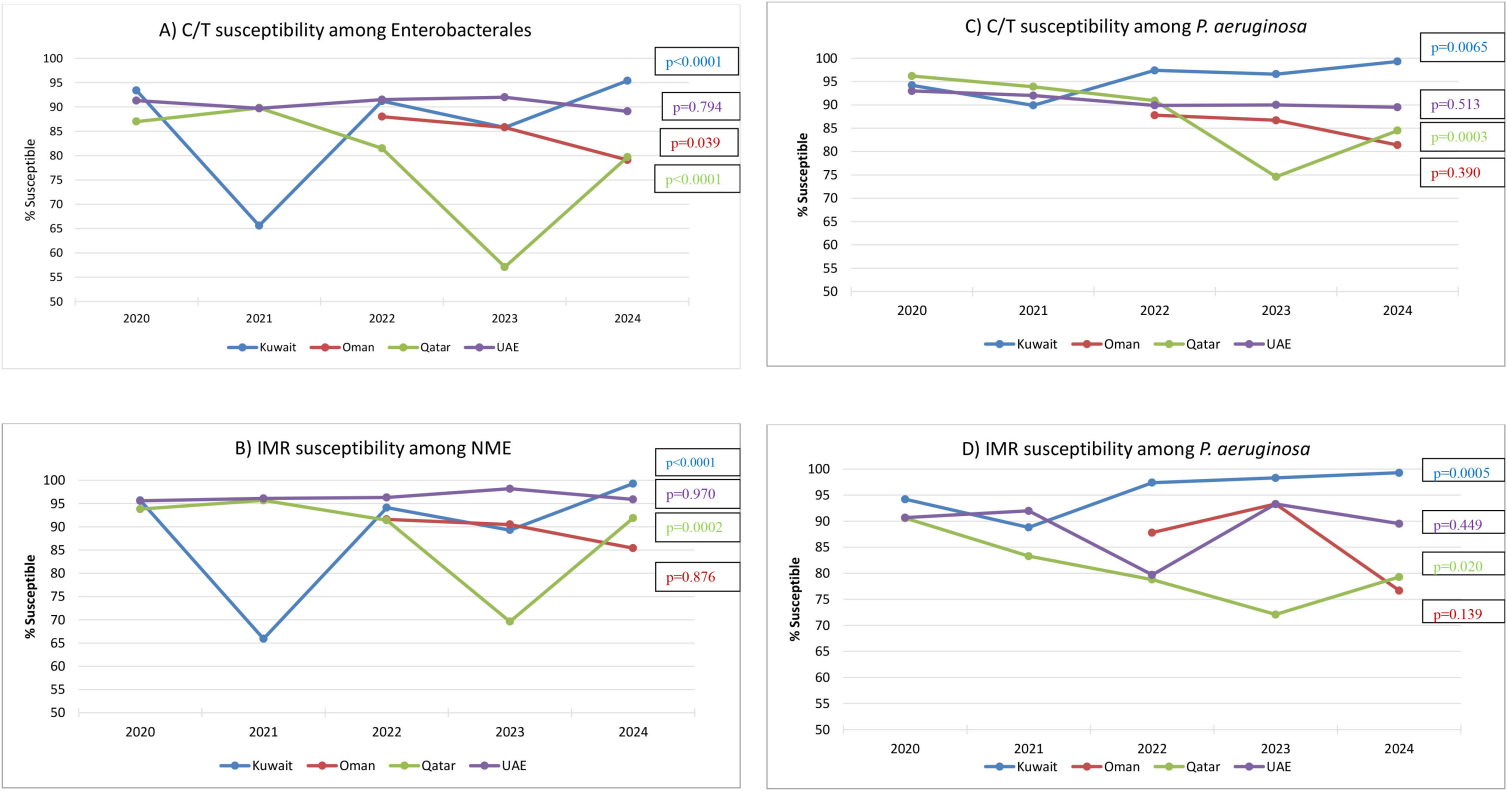
Country-specific annual trends in the susceptibility of **(A)** Enterobacterales to ceftolozane/tazobactam (C/T), **(B)** non-*Morganellaceae* Enterobacterales (NME) to imipenem/relebactam (IMR), **(C)** *Pseudomonas aeruginosa* to C/T, and **(D)** *P. aeruginosa* to IMR. Significance in trend over time determined by the Cochran–Armitage test. A *p*-value <0.05 was considered significant. Percent susceptible values presented in this figure are provided in **Supplementary Table 3**.

Annual trends in country-specific susceptibility showed significant year-to-year variation in some countries, especially among the Enterobacterales and NME (**Figure 2**). Regarding Enterobacterales susceptibility to ceftolozane/tazobactam, the Cochran–Armitage test revealed a highly significant trend of increasing susceptibility among isolates from Kuwait (*p* < 0.0001), but a decreasing trend in Qatar (*p* < 0.0001), while a moderately significant decreasing trend in Oman was observed (*p* = 0.039). Similarly, for imipenem/relebactam against the NME, a highly significant trend of increasing susceptibility was seen in Kuwait (*p* < 0.0001), while a highly significant decreasing susceptibility trend was observed in Qatar (*p* = 0.0002). The same country-specific trends observed for the Enterobacterales/NME extended to *P. aeruginosa* as well. *Pseudomonas aeruginosa* collected in Kuwait exhibited statistically significant trends of increasing susceptibility to ceftolozane/tazobactam and imipenem/relebactam from 2020 to 2024, whereas in Qatar, significant trends of decreasing susceptibility were observed. It should be noted, however, that given the small number of participating medical centers per country (two in Kuwait, one in Qatar), these trends may reflect changes in patient populations, outbreak events, or infection control practices at individual hospitals rather than true national-level trends. Among *P. aeruginosa* from Oman and the UAE, no significant trends in annual susceptibility rates were seen to either agent.

**Figure 3:**
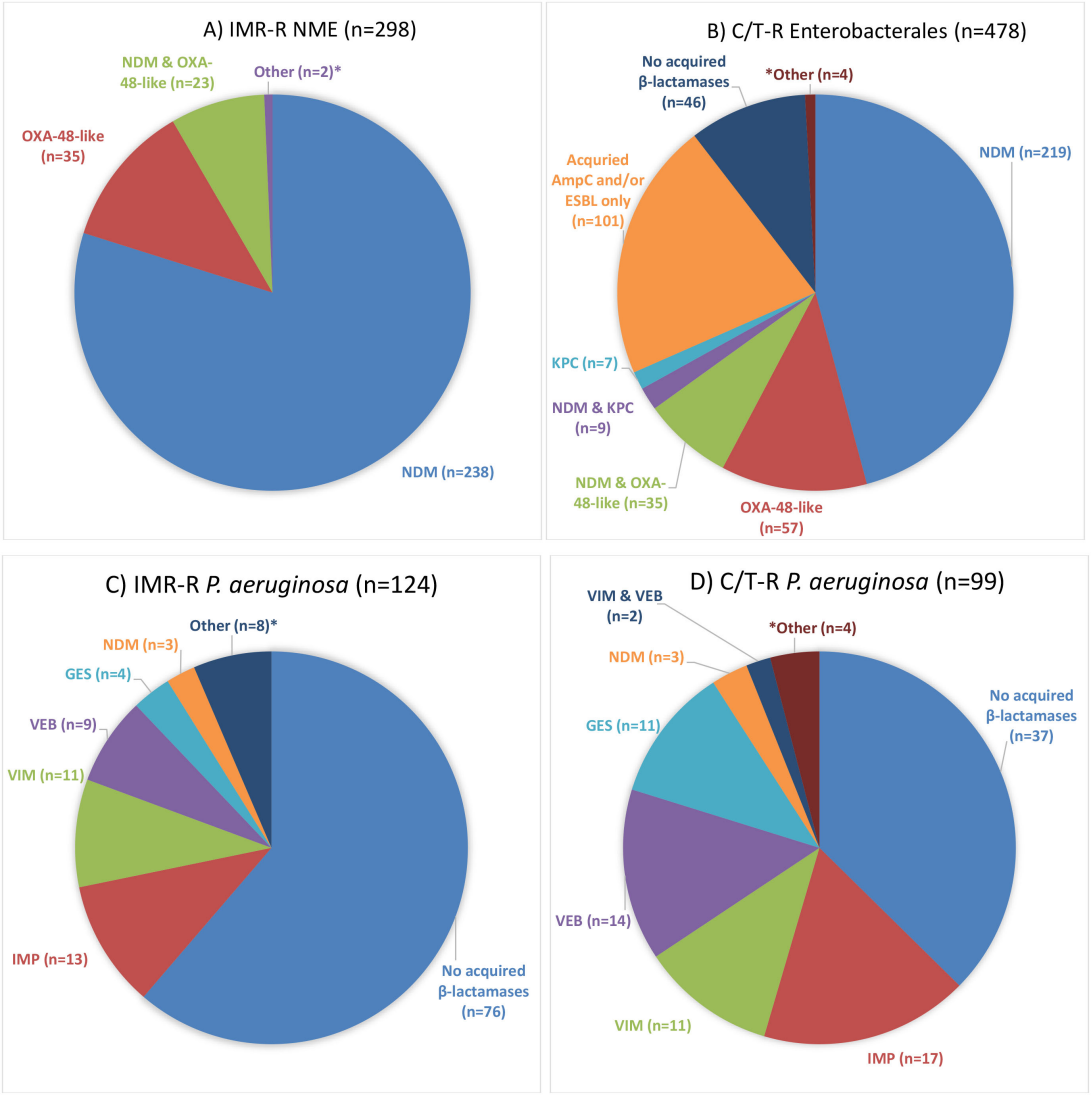
β-Lactamase content among imipenem/relebactam (IMR)- and ceftolozane/tazobactam (C/T)-resistant non-*Morganellaceae* Enterobacterales (NME)/Enterobacterales **(A, B)** and *Pseudomonas aeruginosa* **(C, D)**. * Panel A: VIM (n=1); CTX-M-15 & OXA-1 (n=1). Panel B: KPC & OXA-48-like (n=3); VIM (n=1). Panel C: VIM & VEB (n=2); VIM & IMP (n=1); NDM & DIM (n=1); NDM & VEB (n=1); CMY only (n=1); OXA-14 only (n=2). Panel D: NDM & DIM (n=1); NDM & VEB (n=1); IMP & VIM (n=1); KPC & VEB (n=1).

**Figure 3** summarizes the acquired β-lactamase carriage among imipenem/relebactam- and ceftolozane/tazobactam-resistant (R) NME and Enterobacterales, as well as that of imipenem/relebactam- and ceftolozane/tazobactam-R *P. aeruginosa.* In total, 323 imipenem/relebactam-R NME were identified and 298 (92.3%) were molecularly characterized. The majority, 261/298 (87.6%), carried an NDM metallo-β-lactamase, including 23 that co-carried an OXA-48-like enzyme, while 35 isolates harbored an OXA-48-like enzyme as the sole carbapenemase (**Figure 3A**). Among the ceftolozane/tazobactam-R Enterobacterales (*n* = 520), 478 were characterized (92.0%), and the majority (55.0%; 263/478) carried NDM, including those co-carrying OXA-48-like enzymes (*n* = 35) and KPC (*n* = 9) (**Figure 3B**). Considerable proportions possessed acquired AmpCs and/or ESBLs only, and no carbapenemases (21.1%) or no acquired-β-lactamases at all were detected (9.6%). This latter group consisted primarily of Enterobacterales species expected to possess intrinsic (chromosomal) AmpC-type enzymes, including *Enterobacter* spp., some *Citrobacter* spp., *Klebsiella aerogenes*, and *Serratia* spp.

In total, 124/148 (83.8%) imipenem/relebactam-resistant *P. aeruginosa* isolates were molecularly characterized, and most did not carry an acquired β-lactamase (61.3%), while 32 isolates harbored an MBL, including two multiple MBL carriers [one isolate with VIM and IMP and one with NDM and DIM (Dutch imipenemase)] (**Figure 3C**). The remainder carried ESBLs alone, including VEB (*n* = 9), GES (*n* = 4) or OXA-14 (*n* = 2), or an acquired AmpC (CMY, *n* = 1). Overall, 99/119 (83.2%) ceftolozane/tazobactam-resistant *P. aeruginosa* were characterized. Over one-third, 37/99 (37.4%), did not carry acquired β-lactamases, while 36 (36.4%) carried an MBL, including the two multiple MBL carriers mentioned above (**Figure 3D**). Fourteen isolates harbored VEB alone, while 11 carried GES alone, and one isolate co-carried VEB and KPC.

## 4 Discussion

Although previous reports on Arabian Gulf countries are somewhat limited, recent review articles suggest that there is a substantial burden of resistance in Gram-negative pathogens in the region. For example, a 12-year meta-analysis (2010–2021) of *P. aeruginosa* data from Bahrain, Kuwait, Oman, Qatar, Saudi Arabia, and the UAE showed meropenem resistance ranging from approximately 10% to 46%, with increasing non-susceptibility over time to several anti-pseudomonal agents and the dissemination of high-risk international clones such as sequence type 235 (Alatoom et al., 2024). DTR *P. aeruginosa* phenotypes accounted for approximately 7%–16% of isolates, depending on country and data source (Alatoom et al., 2024), consistent with our present finding of a 7% DTR rate. Regarding Enterobacterales, a recent Gulf Cooperation Council-focused meta-analysis reported that ESBL-producing Enterobacterales account for 21.6% to 29.3% of clinical isolates in the region (Hadi et al., 2023). Molecularly, Gulf ESBL-producing Enterobacterales are dominated by CTX-M (particularly CTX-M-15), with carbapenem resistance largely driven by NDM and OXA-48-like enzymes. These determinants are also common among CRE in global surveillance datasets, including isolates from the Middle East (Lee et al., 2024; Wise et al., 2024). This genetic background has direct implications for the activity of newer β-lactam/β-lactamase inhibitor (BL/BLI) agents, particularly imipenem/relebactam, whose spectrum is limited against class B (MBLs) and OXA-48-like carbapenemases.

Our data from 2020 to 2024 show that ceftolozane/tazobactam retains high *in vitro* activity against *P. aeruginosa* in Arabian Gulf medical centers (91.2% susceptible), including many MDR isolates (62.8% susceptible) and DTR isolates (47.9% susceptible). This is concordant with earlier SMART surveillance results (2017–2020) that showed ceftolozane/tazobactam was active against ~91% of *P. aeruginosa* isolates from the Middle East (Lob et al., 2022), with susceptibility rates that were 14–21 percentage points higher than most other β-lactams, values similar to the current study. An additional Gulf-focused analysis further reported that in Kuwait and Oman, ceftolozane/tazobactam showed greater activity against *P. aeruginosa* than meropenem and piperacillin/tazobactam and was at least as active as amikacin (Alfouzan et al., 2022). Importantly, our data confirm that approximately two-thirds of meropenem-non-susceptible *P. aeruginosa* from the region are susceptible to ceftolozane/tazobactam (66.9%) compared with substantially lower susceptibility percentages to ceftazidime (22.8%) and piperacillin/tazobactam (12.6%). This suggests that, even in settings with high carbapenem resistance, ceftolozane/tazobactam can provide meaningful coverage against *P. aeruginosa*, particularly for non-MBL-producing strains. Ceftolozane/tazobactam resistance is frequently associated with hyperexpression and/or structural mutations in the intrinsic pseudomonal AmpC (PDC) as well as acquisition of carbapenemases and ESBLs (Fournier et al., 2021). The reported high prevalence of MBLs among carbapenem-resistant *P. aeruginosa* in the broader Middle East, therefore, represents a ceiling on ceftolozane/tazobactam’s utility and underscores the need for local enzyme epidemiology to guide empiric use. Overall, however, our findings support positioning ceftolozane/tazobactam as a preferred option for severe infections due to MDR or DTR *P. aeruginosa* in Arabian Gulf centers where MBL prevalence is not overwhelming, especially for hospital-acquired pneumonia and complicated intra-abdominal or urinary tract infections, consistent with current IDSA recommendations (Tamma et al., 2024) and Middle East regional guidelines (Egyptian Drug Authority, 2024). Additionally, the current study showed that ceftolozane/tazobactam performed well against presumed ESBL-producing, non-CRE isolates of *E. coli* (92.0% S) and *K. pneumoniae* (91.6% S). This suggests that ceftolozane/tazobactam may also have utility as a carbapenem-sparing agent against Enterobacterales, once the presence of carbapenemases has been ruled out. Ceftazidime/avibactam, which covered 100% of ESBL, non-CRE *E. coli*, and *K. pneumoniae*, would also be useful in this regard.

Imipenem/relebactam was designed to restore imipenem activity against class A and C β-lactamase producers, including most ESBL- and AmpC-producing Enterobacterales and non-MBL, non-OXA-48 carbapenem-resistant strains, such as those carrying KPC (Zhanel et al., 2018). Global SMART data have shown that adding relebactam increases imipenem susceptibility among Enterobacterales from approximately 92% to 96% overall and restores susceptibility in roughly half of imipenem-non-susceptible isolates (Hilbert et al., 2023). However, imipenem/relebactam is predictably less effective when resistance is driven by MBLs or OXA-48-like enzymes. This has direct implications for Arabian Gulf countries, where OXA-48-like and NDM enzymes have been reported to be the principal carbapenemases in Enterobacterales (Hadi et al., 2023). In the current study, imipenem/relebactam was largely inactive versus CRE (defined phenotypically as resistance to meropenem and/or imipenem), as just 5.6% of the CRE were susceptible. Indeed, molecular characterization of the imipenem/relebactam-resistant CRE confirmed the preponderance of these mechanisms: among the characterized isolates (*n* = 311), 224 (72.0%) and 45 (14.5%) harbored NDM and OXA-48-like carbapenemases, respectively, with 35 (11.3%) carrying both. Nevertheless, current data suggest that imipenem/relebactam can play a targeted role in the region, particularly for ESBL- or AmpC-producing Enterobacterales that are non-susceptible to piperacillin/tazobactam and third-generation cephalosporins but remain without documented MBL or OXA-48-like production, as well as some carbapenem-non-susceptible isolate**s** where strain-specific molecular testing confirms class A carbapenemases (e.g., KPC).

For *P. aeruginosa*, imipenem/relebactam has shown potent activity against many carbapenem-non-susceptible isolates, particularly those with derepressed AmpC and loss of the porin, OprD, while activity is reduced against MBL-producing strains (Mushtaq et al., 2021). Recent clinical studies also report encouraging outcomes for imipenem/relebactam in serious MDR *P. aeruginosa* infections, including pneumonia and bacteremia (Wangchinda et al., 2025). Although Gulf-specific imipenem/relebactam data remain sparse, the underlying resistance mechanisms in regional *P. aeruginosa* isolates (a mix of efflux, AmpC upregulation, and occasional carbapenemases) suggest that a meaningful subset of carbapenem-non-susceptible isolates may become susceptible *in vitro* with imipenem/relebactam.

The strengths of the current study are that it collected a relatively large number of isolates according to a consistent protocol and employed reference broth microdilution antimicrobial susceptibility testing in a central laboratory. However, there are study limitations that should be recognized. Importantly, the small number of medical centers participating in each country (two in Kuwait and one each in Qatar, Oman, and the United Arab Emirates) may not necessarily be representative of the country as a whole, and it is unknown how hospital type, case mix, and the distribution of infection sources could have influenced the results. Similarly, on a regional level, several Gulf countries did not participate in the SMART program (Saudi Arabia, Bahrain, Iran, and Iraq); thus, extrapolation to the entire Arabian Gulf region should therefore be cautious. Budget restrictions allowed for only a subset of collected isolates to be molecularly characterized, and the molecular methods differed for the Enterobacterales over SMART program years. In particular, the target PCR methodology employed in 2020–2022 could fail to detect rarer resistance genes detectable by WGS. Finally, some recently approved antimicrobials, like cefiderocol, were not included on the SMART susceptibility panels. Despite these limitations, the consistency between the present findings and previous reports supports the robustness of the overall message: newer BL/BLI combinations like ceftolozane/tazobactam and imipenem/relebactam represent important additions to the antimicrobial armamentarium against Enterobacterales and *P. aeruginosa* in Arabian Gulf countries. However, their use must be carefully integrated with local resistance mechanisms, diagnostic capabilities, and stewardship priorities.

## Data Availability

The raw data supporting the conclusions of this article will be made available by the authors, without undue reservation.
