# Supplementary material for: Antimicrobial activity of ceftolozane/tazobactam, imipenem/relebactam, and comparators against Gram-negative pathogens collected in Arabian Gulf countries: SMART 2020–2024
Source: Front Cell Infect Microbiol. 2026 May 12;16:1827575. doi: 10.3389/fcimb.2026.1827575 (PMC13201249; doi:10.3389/fcimb.2026.1827575)
Supplement: Supplementary file 1 [file Table1.docx]

SUPPLEMENTARY DATA

**Table S1.** Demographic/clinical characteristics of Gram-negative bacilli collected in Arabian Gulf regions in 2020-2024

|  | | *n* (% of total) | |
| --- | --- | --- | --- |
| Demographic/clinical characteristics | | Enterobacterales^a^  (*n*=3603) | *P. aeruginosa* (*n*=1347) |
| Country | |  |  |
| Kuwait | | 1587 (44.0%) | 589 (43.7%) |
| Oman | | 397 (11.0%) | 114 (8.5%) |
| Qatar | | 761 (21.1%) | 365 (27.1%) |
| United Arab Emirates | | 858 (23.8%) | 279 (20.7%) |
| Year of collection | |  |  |
| 2020 | | 625 (17.3%) | 217 (16.1%) |
| 2021 | | 693 (19.2%) | 205 (15.2%) |
| 2022 | | 750 (20.8%) | 291 (21.6%) |
| 2023 | | 736 (20.4%) | 329 (24.4%) |
| 2024 | | 799 (22.2%) | 305 (22.6%) |
| Infection source | |  |  |
| Bloodstream | | 898 (24.9%) | 149 (11.1%) |
| Intra-abdominal | | 828 (23.0%) | 227 (16.9%) |
| Lower respiratory tract | | 867 (24.1%) | 812 (60.3%) |
| Urinary tract | | 993 (27.6%) | 155 (11.5%) |
| Not specified | | 17 (0.5%) | 4 (0.3%) |
| Patient location | |  |  |
| ICU | | 684 (19.0%) | 327 (24.4%) |
| Non-ICU | | 2658 (73.8%) | 974 (72.3%) |
| Not specified | | 261 (7.2%) | 46 (3.4%) |
| Length of hospitalization at time of specimen collection |  |  |  |
| ≤48 hours | | 1005 (27.9%) | 299 (22.2%) |
| >48 hours | | 2305 (64.0%) | 921 (68.4%) |
| Not specified | | 293 (8.1%) | 127 (9.4%) |

^a^Includes: *Citrobacter amalonaticus (4), Citrobacter freundii (20), Citrobacter koseri (55), Citrobacter sedlakii (5), Citrobacter sp. (9), Enterobacter bugandensis (19), Enterobacter cloacae (91), Enterobacter cloacae complex (9), Enterobacter hormaechei (18), Enterobacter kobei (3), Enterobacter roggenkampii (4), Enterobacter sp. (87), Escherichia coli (1440), Escherichia sp. (11), Klebsiella aerogenes (73), Klebsiella oxytoca (33), Klebsiella pneumoniae (1259), Klebsiella sp. (32), Klebsiella variicola (24), Morganella morganii (40), Proteus hauseri (1), Proteus mirabilis (111), Proteus penneri (2), Proteus sp. (4), Proteus vulgaris (2), Providencia rettgeri (4), Providencia stuartii (18), Raoultella ornithinolytica (1), Raoultella planticola (1), Salmonella sp. (37), Serratia marcescens (87), Serratia nematodiphila (2), Serratia odorifera (1), Serratia rubidaea (1), Serratia sp. (95).*

**Supplemental Table S2.** Susceptibility of Enterobacterales, non-*Morganellaceae* Enterobacterales (NME), and *P. aeruginosa*, to β-lactam antimicrobials by country. Data correspond to Figure 1 in main body of manuscript.

| Organism group, country | Agent, Percent susceptible | | | | | | | |
| --- | --- | --- | --- | --- | --- | --- | --- | --- |
|  | C/T | IMR | IPM | MEM | CZA | FEP | CAZ | TZP |
| Enterobacterales | | | | | | | | |
| Kuwait (n=1587) | 85.8 | NA | 88 | 89 | 89.4 | 63.2 | 57.9 | 79.6 |
| Oman (n=397) | 83.4 | NA | 87.2 | 87.4 | 92.4 | 62 | 54.9 | 79.1 |
| Qatar (n=761) | 80.6 | NA | 88 | 89 | 90.4 | 58.1 | 48.8 | 71.6 |
| UAE (n=858) | 90.7 | NA | 95.5 | 96 | 97.4 | 64.8 | 57.1 | 83.7 |
| NME | | | | | | | | |
| Kuwait (n=1458) | 84.7 | 88.1 | 87.9 | 88 | 88.5 | 60.8 | 55.7 | 78 |
| Oman (n=382) | 83.2 | 88.5 | 87.2 | 87.2 | 92.1 | 61.8 | 54.5 | 78.5 |
| Qatar (n=742) | 80.1 | 89.8 | 88.4 | 88.7 | 90.2 | 57.1 | 48.1 | 71 |
| UAE (n=839) | 90.7 | 96.4 | 96.1 | 96.1 | 97.6 | 64.4 | 56.6 | 83.6 |
| *P. aeruginosa* | | | | | | | | |
| Kuwait (n=589) | 95.9 | 96.1 | 80.1 | 79.3 | 95.4 | 82 | 80 | 75.4 |
| Oman (n=114) | 85.1 | 85.1 | 73.7 | 71.9 | 86 | 74.6 | 71.1 | 70.2 |
| Qatar (n=365) | 85.8 | 79.2 | 49.4 | 44.1 | 78.4 | 53.2 | 49.6 | 44.1 |
| UAE (n=279) | 90.7 | 88.5 | 71.7 | 69.5 | 86 | 65.9 | 64.5 | 62 |

Abbreviations: C/T, ceftolozane/tazobactam; IMR, imipenem/relebactam; IPM, imipenem; MEM, meropenem; CZA, ceftazidime/avibactam; CAZ, ceftazidime; FEP, cefepime; TZP, piperacillin/tazobactam. For IPM against Enterobacterales, the results combine % susceptible, increased exposure values for *Morganellaceae* and % susceptible values for non-*Morganellaceae*. For IPM, CAZ, FEP, and TZP against *P. aeruginosa*, the results represent “% Susceptible, Increased Exposure.” NA, not applicable (no EUCAST breakpoints for IMR against Enterobacterales).

**Supplemental Table S3.** Country-specific annual trends in the susceptibility of Enterobacterales to ceftolozane/tazobactam (C/T), non-*Morganellaceae* Enterobacterales (NME) to imipenem/relebactam (IMR), *P. aeruginosa* to C/T, and *P. aeruginosa* to IMR. Data correspond to Figure 2 in main body of manuscript.

| Organism group, agent country | Year, % Susceptible | | | | |
| --- | --- | --- | --- | --- | --- |
|  | 2020 | 2021 | 2022 | 2023 | 2024 |
| Enterobacterales S to C/T | | | | | |
| Kuwait | 93.4 | 65.6 | 91.2 | 85.8 | 95.4 |
| Oman | - | - | 88 | 85.8 | 79.1 |
| Qatar | 87 | 89.8 | 81.5 | 57.1 | 79.7 |
| UAE | 91.3 | 89.7 | 91.5 | 92 | 89.1 |
| NME S to IMR | | | | | |
| Kuwait | 95.7 | 65.9 | 94.1 | 89.3 | 99.3 |
| Oman | - | - | 91.6 | 90.5 | 85.4 |
| Qatar | 93.8 | 95.7 | 91.4 | 69.6 | 91.9 |
| UAE | 95.6 | 96.1 | 96.3 | 98.2 | 95.9 |
| *P. aeruginosa* S to C/T | | | | | |
| Kuwait | 94.2 | 89.9 | 97.4 | 96.6 | 99.3 |
| Oman | - | - | 87.8 | 86.7 | 81.4 |
| Qatar | 96.2 | 93.9 | 90.9 | 74.6 | 84.5 |
| UAE | 93 | 92 | 89.9 | 90 | 89.5 |
| *P. aeruginosa* S to IMR | | | | | |
| Kuwait | 94.2 | 88.8 | 97.4 | 98.3 | 99.3 |
| Oman | - | - | 87.8 | 93.3 | 76.7 |
| Qatar | 90.6 | 83.3 | 78.8 | 72.1 | 79.3 |
| UAE | 90.7 | 92 | 79.7 | 93.3 | 89.5 |
